# The Impact of Health Insurance Policy on the Fertility Intention of Rural Floating Population in China: Empirical Evidence from Cross-Sectional Data

**DOI:** 10.3390/ijerph20010175

**Published:** 2022-12-22

**Authors:** Yiqing Xing, Clifford Silver Tarimo, Weicun Ren, Liang Zhang

**Affiliations:** 1 School of Politics and Public Administration, Wuhan University, Wuhan 430072, China; 2 College of Public Health, Zhengzhou University, Zhengzhou 450001, China; 3 Department of Science and Laboratory Technology, Dares Salaam Institute of Technology, Dar es Salaam P.O. Box 2958, Tanzania

**Keywords:** health insurance policy, fertility intention, rural floating population, self-selection bias, China

## Abstract

Declining total fertility rates pose a severe challenge to the economy, society, culture, and politics of any region. Low fertility rates among China’s rural floating population with strong fertility are aggravating these challenges. Previous research has confirmed the relationships between health insurance and fertility intention. However, it is still unclear whether the existing association is favorable or not. Moreover, the majority of existing studies in China employ data from either urban or rural populations, whereas evidence from rural floating populations remains scarce. Based on the “China Migrants Dynamic Survey (CMDS)” in 2016, the current study used the logistic regression model to explore the impact of health insurance policy on the fertility intention of the rural floating population in China. Propensity Score Matching (PSM) was used to address potential selection bias. Three important findings were observed: Firstly, participating in the Basic Medical Insurance System (BMISUR) significantly improved rural floating populations’ fertility intentions in China. Secondly, the association between age and the fertility intention of the floating population was “inverted u-shaped” with the highest fertility intention among those aged 25 to 34. There was also a positive correlation between personal income and fertility intention, and it was found between local housing purchase, formal employment, the co-residents scale, and the fertility intention in the rural floating population in China. Interprovincial mobility was positively associated with the fertility intention among rural migrants. Thirdly, the impact of health insurance policies on the fertility intention of the rural migrant population varies by gender, age, and inflow areas. The aforementioned findings can guide the Chinese government in its efforts to improve the fertility intention of the rural floating population, reform the social security system with a focus on “targets”, and implement differentiated welfare policies aimed at promoting the equalization of basic public services, thereby contributing to China’s population structure and long-term development.

## 1. Background

Since the 1970s, the total fertility rate has been declining year by year and has consistently been significantly lower than the birth rate in China since 1990. The proportion is even lower for the floating population (0.94) than for their counterparts living in urban areas (1.28) and rural residents (1.01) [1]. The Chinese population has been considered an “aging population” since the beginning of the 21st century, and the rate of aging is increasing annually. China is currently at a significant risk of falling into a “low-fertility trap” due to its aging population and diminishing birthrate [2]. According to China’s National Bureau of Statistics, China’s birth rate in 2017 was only 12.43 per 1000 people [3]. The rural floating population consists of rural residents who have resided in an urban destination for more than six months away from their local household registry. According to the China Floating Population Development Report of the year 2016, up to 51.1% of the floating population was born in 1980 or later, known as the “new generation of migrants”, and a large proportion is in the fertile period [4]. However, only 12.4% of the floating population of childbearing age have fertility intention, and their low fertility intention is also the new normal [5]. Despite the fact that the Chinese government introduced a universal two-child policy in 2015, meaning the end of the “one-child policy” that has existed in China for 35 years [6]. In 2021, the Communist Party of China (CPC) stated that it would further optimize fertility policy and implement the policy of allowing a couple to have three children [7]. However, the existing research experience has shown that China’s universal two-child policy has not achieved the expected effect of increasing the birth rate [8,9]. It is therefore essential to investigate the factors that influence the fertility intention of individuals of childbearing age. As far as the rural floating population is concerned, the current economic cost element, including the cost of childcare, work opportunity cost, and schooling cost, has been identified as the primary factor influencing their fertility intention [10,11]. At the same time, studies show that future expected returns also affect fertility intention [12]. Existing research has shown that social security acts as a “stabilizer” to resist social risks and improve future expectations [13,14]. However, this effect is limited by China’s long-term implementation of the dual household registration system. Since 1958, the Chinese government has classified Chinese residents into agricultural and non-agricultural groups based on their location (urban or rural) and socioeconomic status (agricultural or non-agricultural), as well as the nature of hukou as the basis of social security distribution [15]. Social security services in many provinces and cities are only provided to the local population, including medical insurance, endowment insurance, unemployment insurance, and others, while the rural floating population is excluded. This also means that, unlike the urban floating population, the rural floating population cannot utilize the public services provided by their place of origin, nor can they gain the social security and social welfare of their point of entry, making them a doubly disadvantaged group. Therefore, is the inadequacy of the health security system among the rural floating population a significant factor in its low fertility rate? A dearth of empirical evidence exists.

Among the diversified and multi-level social security types, the health insurance system has the widest coverage and the greatest impact in China. By the end of 2018, the basic medical insurance for urban and rural residents (BMISUR) covered 1.35 billion people [16]. Therefore, it is representative and of practical significance to conduct research from the perspective of the BMISUR with the characteristics of the rural floating population of childbearing age in a situation where more than 80% of the floating population of childbearing age flow from rural to urban. China’s health insurance system has experienced a gradual development process. In 1998, the Chinese government established the Basic Medical Insurance System for Urban Employees (BMISUE) to meet the needs of China’s market economic reform. In 2003, the Chinese government established the New Rural Cooperative Medical System (NRCMS) to address the health needs of rural residents, which account for more than 50% of China’s population. In 2007, the Chinese government established the Basic Medical Insurance System for Urban Residents (BMISURR) to solve the health coverage of residents who were not covered by BMISUE and NRCMS. In 2015, the Chinese government merged the NRCMS with BMISUR to establish the BMISURR. The health insurance system covers all residents who previously participated in NRCMS and BMISUR. The report to the 19th National Congress of the Communist Party of China (CPC) has proposed to build a multi-tiered social security system that covers the whole population, integrates urban and rural areas, has clear powers and responsibilities, and provides appropriate and sustainable social security, focusing on enhancing fairness, adapting to mobility, and ensuring sustainability.

At present, there are numerous reports on the fertility intention of Chinese residents, focusing on rural women of childbearing age, the urban population [17,18,19], and the entire population of China [20,21]. Limited studies have paid attention to the rural floating population. The studies on the influencing factors of fertility intention mainly focus on the following aspects. First, in terms of personal characteristics. There are gender differences in fertility intention wherein male individuals were shown to have stronger fertility intention than females [22]. Agricultural hukou is positively associated with women’s intention to have two children [23]. Education level has a negative association with fertility intention, the higher the level of education, the greater the employment prospects for women of reproductive age [24], which had a substitution effect on the demands of children, resulting in a decline in fertility intention [25,26]. Some scholars also believe that there is a “hump-shaped” relationship between education level and fertility intention [27]. Secondly, some scholars believe that income level has a negative effect on fertility intention, and the higher the income and status, the more attention paid to the quality of child-rearing and the less the fertility intention [28]. In addition, women with higher employment opportunities have lower fertility intention than women who are not in the labor market [29]. Thirdly, in Chinese society, due to the long-term existence of intergenerational care culture, parents move to the city with their children to help take care of their descendants. Therefore, China also produced the “laopiao” group [30]. In terms of the cost of care, intergenerational rearing can relieve the pressure of women of childbearing age and improve their fertility intention [31]. A preference to have sons is also a typical cultural phenomenon in China and Southeast Asia, and existing experience has shown that selective technology use can lead to a decrease in the number of children and fertility levels based on the child sex perspective [32]. In addition, factors such as external environment and policy affect the fertility intention of the rural floating population. Liu found that Particulate Matter 2.5 has a significant adverse impact on the second-child fertility intention of the floating population [33]. Migration time and experience also affect the fertility intention [34]. It can be seen that the current economic cost is an important factor to consider for the fertility intention of the rural floating population in China.

In addition to current economic costs, in terms of future-expected benefit, existing studies started from the perspective of social security. Moreover, the impact of social security, represented by the health insurance system, on fertility intention is a controversial topic. One viewpoint is that social security has a positive effect on fertility intention. Some studies have shown that social security relieves medical costs and economic burdens of families [35], thereby improving their fertility intention. Meanwhile, due to the limited level of social security protection for floating women and the low expectations of future income, they will increase the number of children in order to achieve security in their later years of life [36]. Another viewpoint argued that social security has a negative effect on fertility intention. Some scholars believe that social security can reduce fertility intention by alleviating the pressure of elderly care [16,37], in which the NRCMS replaced the function of raising children and protecting the old, which reduced the fertility intention of rural residents [38], and the preference for male offspring over females [39]. Moreover, the higher the level of social security, the greater the crowding out effect on fertility intention [40]. It is obvious from the above literature that, although this empirical evidence shows that health insurance policy is closely related to the fertility intention of Chinese residents, several aspects still need to be improved. Firstly, against the background of the diverse internal population structure and special fertility policy in China, relatively few studies have focused on the rural floating population of childbearing age to investigate the individual characteristics, socioeconomic, and mobility factors that may influence their fertility intention. Fertility intention plays an important role in revealing fertility trends, which is an important factor in fertility behavior [41]. Secondly, the complex association between health insurance policy and the fertility intention of migrant women of childbearing age has not been conclusively established and it is still unclear if health insurance policies encourages or discourages fertility intention among the rural migrant population. Finally, the current study on the relationship between social security and fertility intention only performed simple relational regressions, and has not explored the endogeneity of the study, especially the self-selection problem of social security participation, which affects the robustness of the study results [42]. These deficiencies provide direction for the current study.

In summary, current studies have not paid enough attention to the fertility intention of China’s rural floating population, and the research on the relationship between health insurance and the fertility intention of China’s rural floating population has not been explored. In addition, the endogeneity of the relationship between social security and fertility intention has also not been explored. The majority of the rural floating population are in the childbearing age stage, and their fertility intention not only directly affects the implementation effect of China’s fertility policy, but also indirectly affects the fertility intention and behavior of the rural childbearing population of the same age through peer effect, which has an important impact on China’s population structure and long-term sustainable development. In this regard, based on the needs and characteristics of the rural floating population in China, the current study used the data of the China Floating Population Dynamic Monitoring (CMDS) in 2016 to explore the main factors affecting the fertility intention of the rural floating population. It also discusses the relationship between health insurance policies and the fertility intention of the rural floating population. The authors have also used PSM to address the potential selection bias, which will aid in overcoming the possibility of biased findings presented by previous studies on the relationship between fertility intention and social security. In addition, the retrospective design of the current study enhances the reliability and persuasiveness of the research conclusions.

## 2. Materials and Methods

### 2.1. Data Source

The study used the 2016 China Migrants Dynamic Survey (CMDS), which is based on the data of the national floating population in 2015 by the National Health and Family Planning Commission, and obtained via the PPS sampling method of multiple layers and stages. The survey has a total sample size of around 200,600, covers 31 provinces (autonomous regions and municipalities directly under the Central government), and Xinjiang Production and Construction Corps. Participants of the current study were the population of childbearing age at 15–49 years old, with rural household registration and non-local residents. A total of 29,437 eligible samples were obtained after excluding individuals who did not meet the criteria. 

### 2.2. Variables

#### 2.2.1. Dependent Variable

In this study, fertility intention of the rural floating population of childbearing age was taken as the dependent variable. Referring to relevant research on reflecting fertility intention by examining the plans of individuals of childbearing age to have children in a specific period in the future [43,44]. The question “Do you intend to have more children?” available in the 2016 CMDS was used to measure the fertility intention of the rural migrant population of childbearing age. Fertility intention was coded 1–3 indicating “Yes”, “no”, and “uncertain”, respectively. Since the current study researched the rural floating population’s clear fertility intention to have another child, the answer “yes” is assigned a value of 1, indicating the fertility intention to have another child. “No” and “uncertain” are assigned a value of 0, indicating no desire to have more children.

#### 2.2.2. Independent Variable

Health insurance policy was used as the independent variable in this study. As mentioned above, many local governments in China began to explore merging the NRCMS with the BMISUR into the BMISURR in 2015. The 2016 CMDS was investigated from 31 May 2016, just at the merging stage of NRCMS and BMISUR. Therefore, the current study refers to other scholars’ studies [45], and compresses them into one category, and regards participation in any of the types of health insurance as participation in BMISURR. Furthermore, in the 2016 CMDS, more than 80% of the rural floating population participated in the BMISUR, indicating that it is representative and can identify the impact of health insurance policies on the fertility intentions of the rural floating population effectively and reliably.

#### 2.2.3. Controlled Variables 

In addition to health insurance participation, the individual characteristics of the rural floating population of childbearing age include age, age square, sex, and education level. Economic characteristics were also included as control variables, namely personal income, housing property, and employment. Among them, the types of employment were categorized into formal and informal employment. Referring to Maloney’s classification criteria, non-signed labor/service contracts and informal employment relations were regarded as informal employment [46]. In addition, the characteristics of migration variables that may affect the fertility intention of the rural migrant population were also controlled, including migration times, migration range, co-residence scale, and inflow region. The definition of description of each variable is shown in Table 1.

### 2.3. The Model 

#### 2.3.1. Basic Model 

We used “fertility intention” for the rural floating population as the dependent variable. Since the dependent variable is a binary categorical variable of 0–1, we used a Binary Logistic Regression model to evaluate the impact of health insurance policy on fertility intention of the rural floating population. A Binary Logistic Regression model is always set to analyze the relationship between some variables and fertility intention [47]. The basic model is:

$$Y_{i}=\alpha +\beta_{1}\times x_{i}+\beta_{2}\times m_{i}+\varepsilon_{i}$$

where *Y_i_* denotes the probability of the rural floating population’s fertility intention in China, and *x_i_* represents the status of the rural floating population participating in health insurance, *m_i_* indicates a series of control variables, including demographic characteristics, social characteristics, and mobility characteristics, and *ε_i_* is a random disturbance term. We focus on *β*_1_, which reflects the impact of health insurance policy on the fertility intention of the rural floating population. A positive value of *β*_1_ indicates that the participation of the rural floating population of childbearing age in a health insurance policy is likely to improve their fertility intention.

#### 2.3.2. Propensity Score Matching (PSM) 

Participation in health insurance was included as an independent variable in this study. Due to the moral hazard in the health insurance market, that is, whether or not to participate in the insurance is a choice made based on personal reality and not controlled by human factors, it was impossible to randomly restrict to people who participate in health insurance or not. Therefore, whether the sample in the survey participated in the BMISURR or not has the problem of sample selection [48,49], which caused the effect to be over- or under-estimated. The propensity score matching method (PSM) is specifically designed to address sample self-selection bias, which estimates the net effect of health insurance policy on the fertility intention of rural migrants [50,51]. 

PSM analyses are usually divided into three steps. Firstly, propensity scores were estimated using a Binary Logistic Regression model. Based on the above literatures’ review, we set all the controlled variables as independent variables to ensure that the negligibility test is satisfied, and estimated a binary regression model with the fertility intention of the rural migrants as the dependent variable to predict the probability of the rural migrants to participate in health insurance, as shown in Equation (2).

$$Ρ\left(x_{i}\right)\equiv Ρ\left(D_{i}=1|x=x_{i}\right)$$

If the rural floating population participated in the BMISURR, *D_i_* = 1; otherwise, *D_i_* = 0. 

Secondly, propensity score matching was conducted. PSM takes the health insured group as the treatment group and constructs a group of samples with similar characteristics in all aspects, but with differences only in whether to participate in the insurance as the control group. By comparing the differences in fertility intention between the two groups, the size of the effect of health insurance participation and fertility intention of the rural floating population was determined. To ensure the robustness of the results, we adopted four matching methods, namely, nearest neighbor matching, radius matching, kernel matching, and spline matching, and conducted relevant tests [52].

Finally, the average treatment effect (ATT) was calculated. By comparing the difference in fertility intention between the two groups, the effect size of the rural floating population’s participation in BMISURR and fertility intention was determined, as shown in Equation (3).

$$\mathrm{ATT}=E\left(Y_{1i}-Y_{0i}\right)|D_{i}=1$$

Among them, *Y*_1*i*_ and *Y*_0*i*_, respectively, represent the fertility intention of the same floating population in the two different situations of participating in BMISURR and not participating in BMISURR.

## 3. Results

### 3.1. The Logit Regression Model on the Impact of Health Insurance Policy on Fertility Intention of China’s Rural Floating Population 

In order to make the regression results more robust, the method of gradually including control variables was adopted. Table 2 shows the logit regression results of fertility intention of China’s rural migrants of childbearing age. In model 1 to model 6, health insurance on the fertility intention of the rural migrants has a significant synergistic effect, always significantly under the 1% level, and the coefficients were changed in different models. When control variables were not included in model 1, the influence coefficient of health insurance was 0.237 (*p* < 0.01). When demographic variables were included in model 2, the coefficient of health insurance became 0.274 (*p* < 0.01). When socioeconomic variables were included in model 3, the coefficient of health insurance became 0.169 (*p* < 0.01). When demographic and socioeconomic variables were included in model 4, the coefficient became 0.190 (*p* < 0.01). In model 5, when the migration characteristic variables were included, the coefficient become 0.201 (*p* < 0.01). In model 6, when all controlled variables were included, the health insurance coefficient became 0.186 (*p* < 0.01). It can be appreciated that health insurance has a significant and robust effect on the fertility intention of the rural migrants of childbearing age in China.

At the same time, according to the results of model 6 in Table 2, the age and fertility intention of the rural floating population present an “inverted U-shaped” relationship. With the increase in age, the fertility intention of rural migrants rises first and then decreases. The fertility intention of rural migrants between 25 and 34 years old was higher. Gender has a significant impact on the fertility intention of rural migrants. Compared with male migrants, the fertility intention of rural female migrants was lower (coefficient = −0.242, *p* < 0.01). Regarding economic features, there is a favorable correlation between personal income and fertility intention among rural migrants. With an increase in income, the fertility intention of rural floating population grew (coefficient = 0.086, *p* < 0.01). Housing property has a significant impact on the fertility intention of the rural migrants. Compared with renting, buying a house locally significantly improves the fertility intention of the rural migrants (coefficient = 0.079, *p* < 0.01). In terms of employment types, compared with informal employment, formal employment improves the probability of fertility intention of China’s rural floating population (coefficient = 0.078, *p* < 0.05). From the perspective of the migration characteristics, the more migration times, the greater the fertility intention of the rural floating population (coefficient = 0.058, *p* < 0.01). The larger the co-living scale in the inflow area, the greater the fertility intention of the rural floating population (coefficient = 0.055, *p* < 0.05). In terms of mobility range, compared with interprovince migration, the near-distance intercity migration (coefficient = 0.204, *p* < 0.01) and intercounty migration (coefficient = 0.246, *p* < 0.01) leads to higher fertility intention. 

### 3.2. Regression Results of the Impact of Health Insurance Policy on Fertility Intention of Rural Floating Population under Propensity Score Matching 

As mentioned above, we used PSM methods to address self-selection bias in participating in health insurance. On this occasion, we deployed radius matching, kernel matching, and spline matching to test the synergistic effect of participating in health insurance on the fertility intention of the rural floating population by reporting the ATT. As shown in Table 3, under different propensity score matching methods, the coefficient *B* was less than 25, and the *R* value was between 0.25–2. This indicates that the sample data were balanced after matching. Secondly, before using the propensity score matching method, the synergistic effect of participating in health insurance on fertility intention of the rural floating population was 0.179, which is significantly correlated at the level of 1%. After using different matching methods, the treatment effect increased and remained at 0.220, which is still significantly positive at the 1% level, indicating that the synergistic effect of insurance participation on the fertility intention of the rural floating population is robust.

### 3.3. Robustness Test 

In order to test the robustness of the above results, the research processed the dependent variable with different methods, and used a logit regression model and a probit model, respectively, according to different types of dependent variables to test the promoting effect of participating in health insurance on the fertility intention of the rural floating population.

Table 4 shows the regression results of the impact of BMISURR on the fertility intention of rural migrants under different models. Specifically, columns 1 and 2 processed fertility intention into ordered categorical variables, including three categories of “unwilling to give birth, not thinking well, willing to give birth”, and used the regression results of the Mlogit model. The results showed that compared with the rural migrants of childbearing age who were not willing to give birth, participating in BMISURR significantly increased the probability of willing to give birth (coefficient = 0.108, *p* < 0.01). Column 3 still treated fertility intention as a binary variable but used a probit model for regression. Both the logit model and the probit model were suitable for a binary choice model where the independent variable is 0–1, and the occurrence probability of an event depends on the independent variable. The results showed that participation in BMISURR can still significantly improve the fertility intention of the rural migrants of childbearing age (coefficient = 0.237, *p* < 0.01). In conclusion, whether the fertility intention variable is treated as a binary or ordered classification variable, and the probit model or logit model was used, health insurance has a significant synergistic effect on the fertility intention of the rural floating population.

### 3.4. Impact of Health Insurance on Fertility Intention of Rural Floating Population: Age Difference 

The above analysis was about the average effect of the whole sample, and did not take into account the heterogeneity of the impact of health insurance on the fertility intention of the rural floating population. Therefore, we further discussed the age, sex, household, and regional heterogeneity of synergies. To facilitate comparison and account for heterogeneity in the regression results, odds ratios were reported in full instead of coefficients. 

In order to more precisely determine the age gap of synergistic impact, this study split the age of rural floating population into seven age groups with a 5-year age gap, based on the perspective of age difference. Table 5 reports the regression results of health insurance on the fertility intention of rural migrants of different ages in China. The results showed that the health insurance has no significant effect on the fertility intention of rural migrants under 19 years old and over 40 years old. However, it has a positive impact on the fertility intention of the rural floating population aged 20–24 (coefficient = 1.333, *p* < 0.05), and has a positive impact on the fertility intention of the rural floating population aged 25–29 (coefficient = 1.121, *p* < 0.01). Besides, health insurance has a positive impact on the fertility intention of the rural floating population aged 30–34 (coefficient = 1.170, *p* < 0.05), and has a positive impact on the fertility intention of the rural floating population aged 35–39 (coefficient = 1.310, *p* < 0.01).

### 3.5. Impact of Health Insurance on Fertility Intention of Rural Floating Population: Sex and Regional Differences 

Table 6 reports the regression results of the impact of health insurance on the fertility intention of the rural floating population in China by gender and region. According to the regression results by gender, participating in health insurance has a significant impact on males’ fertility intention (coefficient = 1.325, *p* < 0.05), but has no significant impact on females’ fertility intention. According to the regression results by region, participating in health insurance has a positive impact on eastern fertility intention (coefficient = 1.241, *p* < 0.01), and also has a promoting effect on fertility intention for the rural floating population flowing into the western region (coefficient = 1.231, *p* < 0.01). However, health insurance has no effect on the fertility intention of the rural floating population of childbearing age flowing into the central and northeast regions.

## 4. Discussion 

China’s floating population has a large scale and is dominated by young and middle-aged individuals aged 15–49 with high fertility ability [53]. The fertility intention of the rural floating population is not only directly related to the implementation effect of China’s fertility policy, but also can pass the fertility intention to the rural population through peer effect. It is of strategic significance to China’s population structure and labor supply [54]. Based on 2016 CMDS data, this paper used PSM to discuss health insurance participation and the fertility intention of the rural floating population in China, overcoming the estimation bias during sample selection. At the same time, the substitution model and different property variables were used to test the robustness and further study the credibility of the results. In addition, the heterogeneity analysis was carried out from the perspective of age, sex, and inflow regions.

### 4.1. Health Insurance and Fertility Intention of China’s Rural Floating Population 

The results showed that health insurance has a synergistic effect on the fertility intention of rural migrants in China, rather than a crowding out effect. That is to say, participating in health insurance improves the fertility intention of China’s rural floating population, which is related to the nature of the BMISURR. In 2015, Guangdong, Zhejiang, Shandong, Xinjiang, and other provinces in China began to explore the integration of the NRCMS and BMISUR into the BMISURR [55]. In 2016, health insurance consolidation became a national policy. The BMISURR adopts the principle of “Choose wide over narrow, high over low” and covers all residents who previously participated in the NRCMS and BMISUR. Although the level of health insurance payments for rural residents increased, the merger of the two insurances led to the adoption of a broader health care medicine directory, expanded coverage of medical services, and higher reimbursement rates. Therefore, the ability of rural residents to resist risks has been improved [56].

In addition, the BMISURR continues to follow the principle of voluntary participation, combining individual contributions with government subsidies, and the level of government subsidies is constantly increasing. For example, China’s Yunnan province proposed to increase government subsidies to 420 yuan per capita by 2016. Shanghai proposed that the individual contribution should account for 15% of the total financing, the financial subsidy between the city (including the central government) and the county and district should be shared 1:1 in 2016. It can be seen that the BMISURR has a high level of subsidies and reimbursement, which relaxes the family budget constraints of the rural floating population and, to some extent, relieves the economic and psychological pressure caused by childbearing so as to reduce the precautionary saving motivation, improve their current consumption ability, and thus enhance their fertility intention. This is consistent with the view of many scholars [12,40]. This study further found a positive correlation between health insurance and fertility intention among rural migrants in China. This conclusion not only provides a reference for the Chinese government to raise the fertility intention of the floating population but also provides policy direction for other low fertility countries which are similar to China’s stage of development. According to the needs of the rural floating population, the government should constantly improve the level of health care and their expected future income to improve their fertility intention and behavior.

### 4.2. Other Factors Affecting Fertility Intention of Rural Floating Population in China 

According to the results in Table 4, the relationship between age and the fertility intention of the rural floating population in China is relatively complex, showing a trend of increasing first and then decreasing, which is related to time cost, physical risk, economic pressure, and other factors brought about by increasing age, this is consistent with Shi’s findings [57]. Personal income has a positive effect on the fertility intention of China’s rural floating population. The higher the income level, the greater the fertility intention. This finding contradicts recent studies on income level and fertility intention [28]. This may be because childbearing is a decision based on economic considerations, especially for the rural floating population, and low-income level is the main factor limiting their fertility intention [58]. They can get more employment opportunities and higher incomes after entering the city, which reduces the economic pressure brought by childbearing, thus improving their fertility intention. Women are less likely to have children than men. This may be because women are in a weaker position in the labor market, and the economic pressure brought by childbirth has a stronger impact on them, which in turn affects their fertility intention.

Interestingly, this study found that the nature of housing is significantly correlated with the fertility intention of the rural floating population in China. Compared with renting, local housing can improve their fertility intention, which is inconsistent with the research conclusion of Mulder CH [59]. He argued that home ownership causes residents to delay the time to form a family and choose to have children, thus inhibiting the desire to have children. Different from foreign cultures, Chinese culture emphasizes “Only have a house for happy work and life” [60]. For migrants who have left their original places of residence, owning housing property rights means higher social integration and identity, which can improve their life satisfaction and fertility intention [61]. Besides, compared with informal employment, formal employment significantly increases the fertility intention of rural migrants in China, which may be because the stable wage income of formal employment can increase their future income expectation, relax the family budget constraint, and then improve their fertility intention.

In addition, the scale of living together in migrant areas significantly improves the fertility intention of the rural floating population. Family members living together in migrant areas can take care of their children, which greatly reduces the fertility pressure of people of childbearing age and thus improves their fertility intention. This conclusion is related to the recent emergence of the “floating elderly” group in China, who are over 60 years old and continue to follow their children in order to take care of their children and grandchildren [62]. According to data from the China Migrants Development Report 2018, the number of floating elderly in China has increased from 5.03 million to 13.04 million from 2000 to 2015 [63]. In the future, with the continuous promotion of new urbanization and the continuation of intergenerational support, the scale of floating elderly will continue to rise [64]. This also provides a direction for the Chinese government to optimize social security, that is, in order to fully guarantee and enhance the fertility intention of the rural floating population, attention should be paid to the group of elderly people living with them, and improve their level of local medical security and old-age security. Besides, in terms of mobility scope, compared with near-distance mobility, long-distance mobility reduces the fertility intention of the rural floating population. This may be because cross-provincial mobility is faced with greater resource constraints, including children’s care resources and parents’ support resources [64], thus reducing the fertility intention of the rural floating population.

### 4.3. The Impact of Health Insurance Policy on Fertility Intention of Rural Floating Population in China Is Different in Ages, Genders and Regions 

This study found that health insurance has different effects on the fertility intention of rural migrants in different ages. The effect of participating in the BMISURR on the fertility intention is mainly concentrated in the population aged 25–39, and has no effect on the population aged below 19 and above 40. However, the population of childbearing age between 25 and 39 years old is not only at the peak of childbearing behavior, but also in the middle age of supporting the elderly and raising children, with great economic, life, and work pressure. The subsidized BMISURR can boost their future income prospects and alleviate their current pressure. The future trajectory of China’s population structure is heavily influenced by the fertility intention and behavior of various groups. This provides a direction for China’s social security reform, and requires that the reform “target” should focus on the rural floating population aged 25–39.

Secondly, health insurance has varying effects on the fertility intentions of rural floating populations of both sexes. Participation in the BMISURR had a positive influence on male fertility intention, but no effect on female fertility intention. This is basically in line with the characteristics of the floating population and the family division of labor. Young members of the floating population are mainly migrant workers and businessmen, and males bear the main economic responsibility of the family. Health insurance can alleviate their economic strain on the cost of living in their destination and improve their future aspirations, thus increasing their fertility intention. In this sense, China’s medical insurance reform must include measures that are more sensitive to gender differences.

Finally, health insurance has varying effects on the fertility intention of the floating population in various regions. Behind economic restrictions and structural considerations, the fertility goal is reflected from the perspective of the inflow area. The BMISURR has no effect on the fertility intention of the rural floating populations in the central and northeastern areas. On the one hand, although there are more job opportunities and higher living costs in the eastern region, the BMISURR can relieve the pressure of the rural floating population in the eastern region and promote their fertility intention. On the other hand, the cost of living in the western region is low, because the BMISURR is mainly subsidized, it relaxes the family budget and improves the family’s willingness to have children by improving their consumption ability. The results indicated that we must not only consider the fertility intention of the floating population in developed regions, but also the impact of health insurance in developing regions.

This study may contribute to the current literature on fertility intention and influencing factors of the rural floating population in China. Firstly, the majority of research on the fertility intention of Chinese residents focuses on urban and rural populations, but there is relatively limited research on the fertility intention of the rural floating population. This study focused on the rural floating population and explored their fertility intention and influencing factors, which could enrich the research scope on the fertility intention of the Chinese population. Secondly, while there are limited studies on the relationship between social security and fertility intention, current research on the relationship between social security and fertility intention remains controversial. This paper selected the BMISURR that covers the largest range of the rural floating population after the implementation of the two insurance policies in China, and firstly discussed the correlation between health insurance policy and the fertility intention of China’s rural floating population. The findings respond to existing research on the controversial relationship between health insurance and fertility intention, and demonstrate that health insurance policy has a significant positive impact on fertility intention for the rural floating population. Therefore, it fills a gap in the lack of research on fertility intention among the rural floating population. We deployed PSM to address the self-selection bias in health insurance participation. To sum up, the findings of this study not only expand the field and content of current studies, but also enrich the research methods of related content and improve the existing research.

The study has several limitations. Firstly, due to the fact that the latest data did not involve the fertility intention of the floating population, this paper selects a relatively new 2016 CMDS data. To determine the changes in the fertility intention of the floating population in response to the universal three-child policy, it is still essential to analyze the most recent data. Secondly, only the BMISURR with the largest coverage and the widest range of the rural floating population are considered in this paper, no other types of social security, such as urban residents’ medical insurance, endowment insurance, maternity insurance, etc. are considered. Comprehensive inspection of all insurance types for the influence of the rural floating population fertility intention may become more complicated, hence further research is needed in the future.

## 5. Conclusions 

This current study quantified the impact of health insurance on the fertility intention of rural migrants in China using the 2016 CMDS. After addressing the sample selection bias, the health insurance policy has significantly improved the fertility intention of the rural floating population of childbearing age in China. It was found that participation in the BMISURR significantly increased the fertility intention of the rural floating population, with the probability of increasing the fertility intention of 18.6%. Secondly, the factors that affect the fertility intention of the rural floating population also include age, sex, economic income, housing property, employment type, co-living scale, migration times, and range of migration. Thirdly, the BMISURR has varying effects on the fertility intention of the rural floating population of different ages, genders, and regions. These results provide a new perspective for improving the fertility intention of the rural floating population, and also provide ideas on the reform of social security in China. Future studies on fertility intention should focus on the groups with strong fertility ability but fragile fertility intentions and behaviors in a country or a region, as well as the main factors influencing their fertility intention, so as to provide targeted recommendations for improving the local population structure and achieving sustainable development. 

For the Chinese government, the future should be in various areas of reform. Firstly, the Chinese government should adjust and improve the BMISURR, so as to increase the health insurance participation rate of the rural floating population, and improve the future expectation guarantee for the fertility intention of the rural floating population. Secondly, the equalization of basic public services should be promoted for the rural floating population, opening urban public resources, especially community medical services to them, and setting up resources and services for the rural floating population in cities where conditions permit, so as to improve the fertility expectation of the rural floating population. Thirdly, government should pay attention to the elderly who follow their children on the move, and provide high-quality elderly care services and medical services for the floating elderly, so as to ensure an adequate supply of family care services. Fourth, the government should implement differentiated welfare policies. The groups with significant improvement effects should become the target groups for future Chinese government reform, including the new generation aged 20–39, men, and the floating population in the eastern and western regions. Specifically, the influences of health insurance policies can be bolstered by continuously increasing the coverage and reimbursement level of the BMISURR, accelerating the policy of reimbursement for the BMISURR in different regions, and enhancing the level of medical insurance coordination, so as to maintain people’s fertility intention. For rural floating females with a low promotion effect of fertility intention, a series of complementary policies to reduce the cost of childbirth should be implemented, focusing on promoting gender equality in the labor market by implementing flexible working hours, and increasing parenting allowances and housing security allowances, so as to reduce the pressure of their fertility costs and enhance their fertility intention.

## Figures and Tables

**Table 1:** Definitions of variables and description of the mean.

Variable		Definition	Mean	SD	Min	Max
Dependent Variable	Fertility Intention	Dummy variable: yes = 1, no = 0	0.226	0.418	0	1
Independent Variable	Health Insurance	Dummy variable: participated in the NRCMS or BMISUR or BMISURR = 1, otherwise = 0	0.727	0.445	0	1
Demographic Characteristics Variables	Age	Continuous variable: participant’s age (range 15 to 49)	32.670	8.094	15	49
	Age squared	Continuous variable: the squared age of the participant	1133.000	542.5	225	2401
	Gender	Dummy variable: male = 1, female = 0	0.489	0.500	0	1
	Education					
	Lower education level	Dummy variable: primary education level and below =1, otherwise = 0	0.118	0.323	0	1
	Secondary Education level	Dummy variable: secondary school education =1, otherwise = 0	0.477	0.499	0	1
	Higher education level	Dummy variable: higher high school education or above =1, otherwise = 0	0.405	0.491	0	1
Economic Characteristic Variables	Personal income	Continuous variable: log of personal income	8.126	0.609	1.609	11.513
	Housing property	Dummy variable: purchase = 1, otherwise = 0	0.249	0.433	0	1
	Employment	Dummy variable: regular employment = 1, informal employment = 0	0.669	0.471	0	1
Migration Characteristic Variables	Migration times	Continuous variable: participant’s migrated frequency and times	1.346	1.007	1	36
	co-residents scale	Continuous variable: participant’s living together, number in the local area	3.105	1.132	1	10
	Range of Migration					
	Interprovince	Dummy variable: participant migrated from one province to another = 1, otherwise = 0	0.492	0.500	0	1
	Intercity	Dummy variable: participant migrated from one city to another within the same province = 1, otherwise = 0	0.335	0.472	0	1
	Intercounty	Dummy variable: participant migrated from one county to another within the same city = 1, otherwise = 0	0.173	0.377	0	1
	Region of Migration	Categorical variable: Eastern = 1, Central = 2, Western = 3, Northeast = 4	2.054	1.015	1	4

**Table 2:** Logit regression results of the impact of Health insurance on fertility intention of China’s rural floating population.

Variables	Regression					
	(1)	(2)	(3)	(4)	(5)	(6)
Health insurance	0.237 ***	0.274 ***	0.169 ***	0.190 ***	0.201 ***	0.186 ***
	(0.022)	(0.025)	(0.032)	(0.035)	(0.022)	(0.035)
Age		0.323 ***		0.332 ***		0.329 ***
		(0.017)		(0.028)		(0.028)
Age squared		−0.007 ***		−0.007 ***		−0.007 ***
		(0.000)		(0.000)		(0.000)
Gender		−0.235 ***		−0.255 ***		−0.242 ***
		(0.020)		(0.034)		(0.032)
Secondary education level		−0.023		−0.033		−0.046
		(0.045)		(0.074)		(0.074)
Higher education level		0.007		0.022		−0.010
		(0.046)		(0.077)		(0.077)
Personal income			0.169 ***	0.058 *		0.086 **
			(0.029)	(0.033)		(0.034)
Housing property			0.077 **	0.064 *		0.079 ***
			(0.033)	(0.035)		(0.036)
Employment			0.115 ***	0.071 **		0.078 **
			(0.034)	(0.036)		(0.036)
Migration times					0.019 **	0.058 ***
					(0.010)	(0.014)
Co-residents scale					0.272 ***	0.055 **
					(0.017)	(0.027)
Intercity					0.149 ***	0.204 ***
					(0.023)	(0.037)
Intercounty					0.118 **	0.246 ***
					(0.029)	(0.047)
Region of migration	Control	Control	Control	Control	Control	Control
n	29,437	29,437	29,437	29,437	29,437	29,437
Pseudo-R^2^	0.0118	0.1019	0.0135	0.0890	0.0271	0.0914

Note: In models 2, 3, 4, 5, and 6, the baseline variable for “participation in BMISURR” is no participation. The baseline variables for gender, housing property, and employment type are male, renting, and informal employment. *, **, *** indicate significance at levels of 10%, 5%, and 1%, respectively. In parentheses are robust standard errors.

**Table 3:** Balance test and average treatment effect of matched samples.

Matching Method	PsR^2^	*p* > chi2	Mean Bias	Med Bias	*B*	*R*	ATT	T
Unmatched	0.224	0.000	31.9	18.0	123.1 *	0.91	0.179 ***	5.08
Nearest neighbor matching	0.003	0.000	2.4	2.0	12.1	1.00	0.220 ***	5.80
Radius matching	0.003	0.000	2.7	2.2	11.9	0.98	0.220 ***	5.80
Kernel matching	0.003	0.000	2.8	2.9	12.4	1.07	0.220 ***	5.66
Spline matching	0.003	0.000	2.9	2.9	13.4	0.94	0.220 ***	4.66

Note: In this table, the nearest neighbor matches 1:4; kernel matching uses the default kernel function with broadband. *, *** indicate significance at levels of 10%and 1%, respectively. In parentheses are robust standard errors.

**Table 4:** Results of the robustness test.

Variables	Mlogit Model		Probit Model
	Uncertain	Willing to Give Birth	
Health insurance	0.095 ***	0.108 ***	0.237 ***
	(0.035)	(0.020)	(0.040)
Other variables	Control	Control	Control
Region of migration	YES	YES	YES
n	29,437	29,437	29,437
Pseudo-R^2^	0.1274	0.0915	0.1274

Note: In this table, *** indicate significance at levels of 1%. In parentheses are robust standard errors.

**Table 5:** Results of age heterogeneity.

Variables	Classification of Age (Year)						
	15–19	20–24	25–29	30–34	35–39	40–44	45–49
Health insurance	3.133	1.333 **	1.121 ***	1.170 **	1.310 ***	1.132	1.610
	(3.910)	(0.171)	(0.069)	(0.073)	(0.120)	(0.182)	(0.606)
Other variables	YES	YES	YES	YES	YES	YES	YES
Region of migration	YES	YES	YES	YES	YES	YES	YES
n	59	1662	8227	7927	5002	4057	2503
Pseudo-R^2^	0.1581	0.0154	0.0124	0.0194	0.0344	0.0414	0.0357

Note: In this table, **, *** indicate significance at levels of 5%, and 1%, respectively. In parentheses are robust standard errors.

**Table 6:** Results of sex and regional heterogeneity.

Variables	Classification of Sex		Classification of Region			
	Male	Female	Eastern	Central	Western	Northeast
Health insurance	1.325 ***	1.079	1.241 ***	1.120	1.231 ***	1.275
	(0.062)	(0.058)	(0.058)	(0.110)	(0.089)	(0.317)
Other variables	YES	YES	YES	YES	YES	YES
Region of migration	YES	YES	YES	YES	YES	YES
n	16,417	13,020	15,418	4250	7542	2227
Pseudo-R^2^	0.0921	0.0942	0.0880	0.0730	0.0847	0.1269

Note: In this table, *** indicate significance at levels of 1%. In parentheses are robust standard errors.

## Data Availability

Data from the China Migrants Dynamic Survey (CMDS) conducted by the National Health and Family Planning Commission were used in this study. (http://www.ldrk.org.cn/ (accessed on 29 May 2020).
